# Prevalence of sexual coercion and its association with unwanted pregnancies among young pregnant females in Kampala, Uganda: a facility based cross-sectional study

**DOI:** 10.1186/s12905-015-0235-9

**Published:** 2015-09-24

**Authors:** Suzan Tusiime, Geofrey Musinguzi, Benjamin Tinkitina, Norah Mwebaza, Rose Kisa, Ronald Anguzu, Noah Kiwanuka

**Affiliations:** School of Public Health Makerere University College of Health Sciences, P.O BOX 7072, Kampala, Uganda; School of Biomedical Sciences Makerere University College of Health Sciences, P.O BOX 7072, Kampala, Uganda

**Keywords:** Sexual coercion, Unwanted pregnancy, Young women, Lubaga Division, Uganda

## Abstract

**Background:**

Sexual coercion is associated with sexually transmitted infections and unwanted pregnancies with consequential unsafe abortions and increased maternal morbidity and mortality. Current literature focuses mainly on its risk factors but less on its resultant deleterious health effects. We conducted a study to determine the prevalence of sexual coercion and its association with unwanted pregnancies among young pregnant women.

**Methods:**

In a cross-sectional study, four hundred and sixteen (416) consenting pregnant females aged 15–24 years attending antenatal clinics in Lubaga division Kampala district in Uganda were enrolled using systematic sampling. Quantitative and qualitative data on sexual coercion were collected by female interviewers.

Adjusted Prevalence Proportion Ratios (Adj. PPRs) of unwanted pregnancy and associated 95 % confidence intervals were estimated by generalized linear models with log link function and Poisson family distribution using robust variance estimator. Quantitative data were analyzed using Stata version 10.0, while qualitative data were analyzed using manifest content analysis.

**Results:**

Prevalence of sexual coercion was 24 % and was higher among those who had non consensual sexual debut (29.0 %) compared with those who had consensual sexual debut (22.6 %). The prevalence of unwanted pregnancy was 18.3 % and was higher among participants who had been sexually coerced relative to their counterparts (*p* < 0.001). History of sexual coercion in the past 12 months and non consensual sexual debut were associated with unwanted pregnancy [adj.PPR = 2.23, 95 % CI: (1.49-3.32)] and 1.72, 95 % CI: (1.16- 2.54)] respectively. Qualitative results indicated that different forms/contexts of sexual coercion, such as deception, transactional sex and physical force influenced unwanted pregnancies.

**Discussion:**

This study highlights that a quarter of our participants in our quantitative study had experienced sexual coercion in the past twelve months and nearly a third of these, had history of non consensual sexual debut. Unwanted pregnancy was higher among the sexually coerced and those who had non consensual sexual debut.

**Conclusion:**

Sexual coercion among pregnant women aged 15–24 years in Kampala, Uganda is high and is significantly associated with unwanted pregnancy. Comprehensive sex education targeting young people (<25 years), along with availability and access to youth friendly centers may be useful in addressing sexual coercion and its negative outcomes.

## Background

Sexual violence is a common problem globally and it comprises of a range of sexual abuse including sexual coercion [1]. Sexual coercion is both a public health problem and a violation of human rights [2]. Globally at least one in three women has been sexually coerced in her lifetime [3]. Young women are predominantly vulnerable to forced sex [4]. In Virginia, USA, 30 % of young women aged 18–24 suffered from rape in 2009 [5]. In Sub Saharan Africa, evidence suggests that between 15- 68 % of young people encountered at least one experience of sexual coercion [6–10] and Uganda particularly experienced up to 67 % [11].

**Fig. 1:**
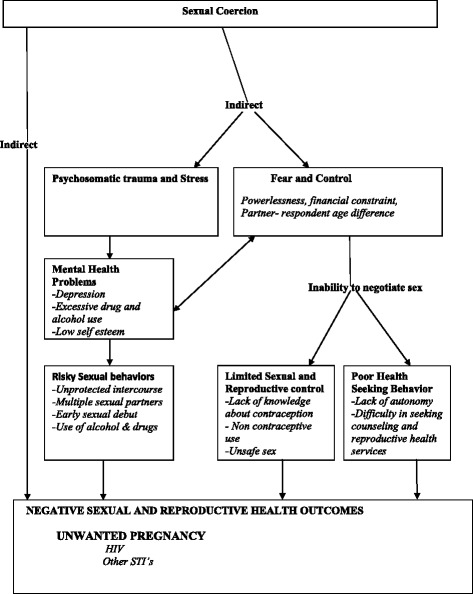
Conceptual Framework. Adapted and modified from WHO [54], Heise and Gottemoeller [17]

A recent national survey in Uganda revealed that up to 36 % of females aged between 15–24 years had ever experienced sexual coercion [12]. Among young women in Uganda, sexual coercion manifests mainly as non-violent coercive sex, unwanted non penetrative touching, verbal harassment, transactional sex and forced sex [11]. In rural Uganda, varying rates of sexual coercion ranging from 22 % in Rakai [9] to as high as 40 % in Mbarara [13] have been reported among young women. Moreover young women are at a higher risk of sexual coercion compared to young boys because of the different gender expectations [2, 14]. Women are not supposed to show sexual interest and boys/men are aware of this and do not respect a woman’s no [14, 15]. In addition, its believed that men are unable to control their sexual desires [8]. Instead, they are supposed to be decision makers in sexual relations and women should comply. Such gender specific expectations promote sexual coercion among women [15–17]. Young women are more vulnerable to the negative reproductive health outcomes of sexual coercion like; unwanted pregnancy, abortion, HIV/AIDS and sexually transmitted diseases [2]. Sexual coercion is associated with unwanted pregnancy [18, 19]. Every year, 14 million unintended (unwanted and mistimed) pregnancies occur in Sub-Saharan Africa [20]. The 2011 Uganda Demographic and Health Survey reported the prevalence of unwanted pregnancy at 40 % [21] and it was put at 80 % in a study at the abortion clinic in Mulago, Kampala [22]. Unwanted pregnancies among young women are associated with school dropout [23], unsafe abortion, child abuse, child morbidity and mortality [24]. About 10 % of young women in Uganda drop out of school due to pregnancy [25], 60,000 die due to problems related to pregnancy and child birth every year [26] and a quarter of unsafe abortions occur among those aged (15–19) years in Africa [27]. Unsafe abortion among young women is a major cause of maternal morbidity and mortality especially in developing countries [28]. However, not all unintended pregnancies are aborted, a few result in live birth including those which were a product of sexual abuse [29]. This happens due to financial constraints [30], fear of negative effects of unsafe abortion or use of non successful methods of abortion [31]. As a result the unwanted pregnancy is carried to term. This exposes both the mother and the baby to a higher risk of morbidity and mortality [24]. Sexual coercion and unwanted pregnancy are common problems among young women in Uganda [32]. “Due to the nature of coerced sex, young women are unable to use a method of contraception” [19] which puts them at risk of unwanted pregnancy. Such pregnancies either result in abortion or unwanted Pregnancies. Both pregnancy and motherhood among young women contribute to the high maternal and child morbidity and mortality [21]. Notably, complications related to pregnancy and child birth are the number one killer of young women more especially those aged 15–19 years [33]. Children born to such young mothers have higher likelihood of death within the first month of life because of malnutrition plus other childhood illnesses [26]. The prevalence of sexual coercion and its association with unwanted pregnancies among young women is not well documented in Urban Uganda. Most previous studies among young people in Uganda have focused on sexual coercion and the associated factors [9, 11, 13, 19, 34, 35] and less on its negative reproductive health outcomes [9, 18]. In this study, we therefore set out to assess the prevalence of sexual coercion and its association with unwanted pregnancy among young pregnant women in an urban setting at health facilities in Uganda with the help of a conceptualized framework Fig. 1. This was a mixed methods study (quantitave and qualitative).

## Methods

### Study setting

This study was conducted in Lubaga division one of the five administrative divisions in Kampala district, Uganda’s capital city. Lubaga was randomly selected out of the five using ballot papers. The division has 58 health facilities of which only 15 offer antenatal services. Nine health facilities with the highest antenatal attendances were purposively selected for inclusion in the study. These included: two public health facilities, two private not for profit hospitals and five private clinics. This study was conducted in antenatal clinics for pragmatic reasons and because of resource constraints. Nevertheless about 98 % of pregnant women in Kampala receive antenatal care from a skilled provider [21] hence we expected to get majority of the study population. We anticipated that young women who were attending antenatal clinics had decided to carry their pregnancies to term including those which were unwanted. From this reason, we were able to estimate the magnitude of unwanted pregnancies that may be resulting from sexual coercion which the young women had carried to term.

### Study design and sampling procedures

This was a facility based cross-sectional study that employed both quantitative and qualitative methods of data collection. We combined both quantitative and qualitative methods of data collection because mixed methods would provide strengths to answer our research question. We aimed at assessing the relationship between sexual coercion and unwanted pregnancy so mixed methods was deemed suitable. The methods gave us a deeper understanding of the complex relationship. Quantitative surveys were administered to 416 young pregnant women aged 15–24 years. The sample size was determined using the Kish Leslie formula [36] a p of 0.36, power of 80 %, alpha of 0.05 and a non response rate of 15 % [22]. Probability proportionate to size sampling was used to select numbers needed at each health facility and systematic sampling to select the final respondent from each health facility.

For qualitative data, eight in-depth interviews (IDIs) were conducted among young women who were purposively selected upon affirmation of a history of both sexual coercion and unwanted pregnancy at the time of conception of the current pregnancy in the quantitative study. Two of the in-depth interview participants had attained pre-secondary education; five had reached secondary while one was at the university. Three of the IDI participants were aged between 15–19 years, whereas five were aged between 20–24 years. Eight key informant interviews (KIIs) were also carried out. Key Informants were purposively selected and these comprised of heads of the youth friendly services selected from each of the eight health facilities except one which lacked any youth programs/youth friendly clinic. The Key Informants were selected because of their knowledge and their role of provision of youth friendly services. Seven of the key informant participants were females. Five of these participants were nurses while three were counselors.

### Data collection and measurement

Quantitative and qualitative data were collected by one of the lead investigators (TS) and 4 trained research assistants from 2nd April to 24th May 2012. Female data collectors were used because of the sensitive nature of the subject matter. Prior to data collection, study tools were pretested for validation in an area outside the study site. A semi-structured questionnaire was used to measure: demographic characteristics, knowledge and use of contraceptives, sexual debut, partner characteristics, youth friendly service related factors, sexual coercion as well as experiences and circumstances surrounding first sex. The IDI’s explored the young women’s lived experience of sexual coercion, how they felt on discovering they were pregnant, their understanding of the causes of unwanted pregnancies and their thoughts about prevention approaches to sexual coercion and unwanted pregnancies. The KII’s were used to provide more understanding on the link between sexual coercion and unwanted pregnancies plus prevention approaches as obtained from their vast experience of working with young people. Qualitative interviews were conducted by TS. All interviews were tape recorded after obtaining consent from the participants. All IDIs were conducted in Luganda (local language) except one. Meanwhile, all KII’s were conducted in English. Each key Informant interview was conducted for about 45 minutes and IDI’s for about 90 minutes. No compensation was provided to participants.

### Definition of variables

#### Unwanted pregnancy

Unwanted pregnancy was our primary outcome and it was defined as a pregnancy that a participant reported of her own free will, as undesired by her [37]. “Unwanted pregnancy” was measured using the following statements in form of a question: “at the time of conception of this pregnancy, “did you want to get pregnant at that time” “did you want to get pregnant later in future” or “you did not want to get pregnant at all?” [38]. The responses included (1 = I did not want to get pregnant at all, (2) = I wanted to get pregnant at that time, (3) = I wanted to get pregnant later in future. Participants who answered that they did not want to get pregnant at all at the time of conception of the pregnancy were taken to have had an unwanted pregnancy.

### Sexual coercion

Sexual coercion was our secondary outcome and it was defined based on the definition by Heise et al. [39] as the act of forcing (or attempting to force) another individual through violence, threats, verbal insistence, deception or economic circumstances to engage in sexual behavior against the individual’s will. Accordingly, we measured sexual coercion using the following set of questions as applied with modification by Wagman et al. [11]. In the past twelve months has any of your sexual partners done the following to you? i) “Forced you to perform other sexual acts you did not want to”; ii) “Used verbal threats to force you to have sex when you did not want to”; iii) “Physically forced you to have sex when you did not want to”; iv) “Forced you to have sex by giving you money or gifts in exchange for sex”; v) “Forced you to have sex through promises the man did not intend to keep such as marriage or extravagant gifts”; If one responded “yes” to at least one of the above, the response was classified as sexual coercion in the past twelve months.

### Circumstance surrounding sexual debut

This was defined as the first sexual intercourse reported by the respondent and the types (consensual and non consensual) were assessed as follows: the first time you had sex, were you forced to have sex? Did you both agree? Or did you force your partner to have sex? When a participant was forced to have sex, we defined the variable as non consensual or otherwise.

Knowledge of contraception: was defined as; having ever heard about contraceptives, naming a source and at least one method of contraceptives. The variable was classified into; knowledgeable (when participants scored one or more), and no knowledge (when the participant scored zero) [40].

### Ethical consideration

Ethical approval for the study was obtained from the Higher Degrees Research and Ethics Committee of Makerere University School of Public Health. The WHO ethical recommendations for conducting research on domestic violence against women were followed [41]. Female research assistants were trained on sexual coercion, the use of a non judgmental attitude while collecting data, how to recognize and respond to participants with emotional changes as well as to refer those who needed help. Prior to the study, health facilities were identified where participants who needed help could be referred. Interviews were conducted under confined rooms or restrained space to ensure confidentiality. All participants provided written informed consent. Females who were married or pregnant and below the age of 18 years were treated as emancipated minor according to the Uganda National guidelines for research involving humans as research participants [42].

### Data analysis

#### Quantitative data

Data were double entered into Epi-Info version 3.5.1 software, cleaned and then exported to Stata version 10.0 for analysis. Covariates were summarized using frequencies for categorical variables while means, standard deviations (SD), medians and inter-quartile range-(IQR) were used for continuous variables. The main outcome was unwanted pregnancy. Unadjusted and adjusted Prevalence Proportion Ratios (PPRs) of unwanted pregnancy and associated 95 % confidence intervals were estimated by generalized linear models with link (log) and family (Poisson) using robust variance estimator [43, 44]. This was done because the proportion of the outcome was greater than 10 % in which case odd ratios would provide biased estimates of associations [45].

Selection of covariates for inclusion in the multivariable model was based on variables found to be predictive of unwanted pregnancy at bivariate analyses at *p* < 0.15 and also using biological plausibility as well as evidence in the empirical literature. Logical model building was done by introducing one variable at a time in the model. Only those variables which retained their significance at *p* < 0.05 and potential confounders which included, knowledge of contraceptives, contraception use at the time of conception and level of education were retained in our final model.

### Qualitative data

Qualitative analyses were done using manifest content analysis. Data were transcribed, read thoroughly line by line and coded using deductive coding. A coding framework was generated using an excel spread sheet. Data were analyzed for content guided by the objectives of the study. Corresponding data were summarized with supporting quotes and presented verbatim.

## Results

### Characteristics of participants

Table 1 shows the descriptive characteristics of study participants. Of the 426 participants approached, 416 (97.7 %) were enrolled and 10 (2.3 %) were not enrolled due to ineligibility or unwillingness to provide written informed consent. Among the enrolled, the mean (SD) and median (IQR) age were 21.2 (2.1) and 21 (20–23) respectively. Majority (76.7 %) were aged 20–24 years, and more than half (52.6 %) were carrying their first pregnancy. Majority of the respondents were Catholics (35.6 %), had attained post primary education (81.3 %), married (80.3 %) and knowledgeable about contraceptives (72.8 %). Forty percent (39.7 %) had their sexual debut between 15–17 years and 22.4 % had non-consensual sexual debut. Twenty one percent had had three or more lifetime sexual partners.

**Table 1:** Selected descriptive characteristic of pregnant women aged 15–24 years attending antenatal clinics in Lubaga, Uganda, 2012

Individual factors	N (%)
Age^†^	
15-19	97 (23.3)
20-24	319 (76.7)
Religion	
Protestant	94 (22.6)
Catholic	148 (35.6)
Muslim	114 (27.4)
Others^**^	60 (14.4)
Level of education	
Pre secondary	78 (18.8)
Post primary	338 (81.3)
Current marital status	
Married^®^	334 (80.3)
Unmarried^††^	82 (19.7)
Knowledge of contraceptives	
No knowledge	113 (27.2)
Knowledgeable	303 (72.8)
Ever used contraceptives	
No	256 (61.5)
Yes	160 (38.5)
Contraception use at the time of conception	
No	364 (87.5)
Yes	52 (12.5)
Age at sexual debut	
18+ years	197 (47.1)
15-17 years	165 (39.7)
< 15 years	23 (5.5)
Unknown	32 (7.7)
Partner respondent age difference at sexual debut	
Partners with the same age or younger	52 (12.5)
Partner 1–5 years older	174 (41.8)
Partner >5 years older	112 (26.9)
Unknown	78 (18.8)
Type of sexual debut	
Consensual	323 (77.6)
Non consensual	93 (22.4)
Lifetime sexual partners	
1-2	329 (79.1)
3+	87 (20.9)
Ever counseled on contraceptives at HCF	
No	267 (64.2)
Yes	149 (35.8)
Ever counseled on sexual violence at HCF	
Yes	96 (23.1)
No	320 (76.9)
Partners level of education	
Low education	33 (7.9)
Secondary	200 (48.1)
Tertiary	116 (27.9)
I don’t know	67 (16.1)
Partner respondent age difference at time of study	
Male partner less than 10 years older	306 (73.6)
Male partner younger or same age	12 (2.9)
Male partner > = 10 years or older	65 (15.6)
Unknown	33 (7.9)
Number of pregnancies before the current	
Prime gravid^♦^	219 (52.64)
1	117(28.13)
2+	80 (19.23)

Age^†^ range is 16–24, mean age (SD) 21.2 (2.1) and median (IQR) 21 (20–23). Others^**^ in religion refer to Pentecostals, Muslims and Orthodox. Unmarried ^††^ includes, single, divorced, widowed and separated. Married^®^ encompasses cohabiting and married in church. Prime gravid^♦^ means this is the first pregnancy she has never had any other before this

### Prevalence of sexual coercion and unwanted pregnancy

The prevalence of sexual coercion (Table 2) was 24 % and was higher among: participants who had non consensual sexual debut (29.0 %) compared with those who had consensual sexual debut (22.6 %); women aged 15–19 years (29.9 %) compared with women aged 20–24 years (22.3 %); those with pre-secondary education (35.9 %) compared to women with Post primary education ; those who had sexual debut before 15 years (52.2 %) compared to those who had theirs at 18 years and above (19.4 %); the unmarried (36.6 %) compared with the married (21 %); women whose male partners were aged 10 years older and above (32.3 %) compared with those whose male partners were in other age categories. The prevalence of unwanted pregnancy was 18.3 %.

**Table 2:** Prevalence of sexual coercion in past twelve months among young pregnant women in Lubaga, Uganda, 2012

Variable	Sexual coercion in the past twelve months % (n/N)
Sexual coercion in the past twelve months	24 (100/416)
Age	
15-19	29.9 (29/97)
20-24	22.3 (71/319)
Level of education	
Pre secondary	35.9 (28/78)
Post primary	21.3 (72/338)
Current marital status	
Married^®^	21.0 (70/334)
Unmarried^††^	36.6 (30/82)
Ever counseled on sexual violence	
Yes	20.8 (20/96)
No	25.0 (80/320)
Age at sexual debut	
18+ years	19.4 (38/196)
15-17 years	24.9 (41/165)
< 15 years	52.2 (12/23)
Unknown	28.1 (9/32)
Type of sexual debut	
Consensual	22.6 (73/323)
Non consensual	29.0 (27/93)
Partner respondent age difference at sexual debut	
Partner with same age or younger	17.3 (9/52)
Partner 1–5 years older	20.7 (36/174)
Partner >5 years older	27.7 (31/112)
Unknown	30.8 (24/78)
Partner respondent age difference at time of study	
Partner <10 years older	22.6 (69/306)
Partner same age or younger	16.7 (2/12)
Partner > =10 years older	32.3 (21/65)
Unknown	24.2 (8/33)
Life time sexual partners	
1-2	21.9 (72/329)
3+	32.2 (28/87)

In Table 2 we have used row percentages that’s why the column percentages do not add up to one hundred. Unmarried ^††^ includes, single, divorced, widowed and separated. Married^®^ encompasses cohabiting and married in church

### The unadjusted and adjusted prevalence proportion ratio of unwanted pregnancy

Table 3 shows that the unadjusted Prevalence Proportion Ratio of unwanted pregnancy was higher among; the sexually coerced [PPR = 2.84, 95 % CI: 1.92-4.20] and young women who experienced non-consensual sexual debut [PPR = 1.81, 95 % CI: 1.19-2.73]. Table 3 further reveals that the unadjusted Prevalence Proportion Ratio of unwanted pregnancy was higher among the unmarried [PPR = 3.48, 95 % CI: 2.37-5.09]. On the other hand, unwanted pregnancy was lower among women aged 20–24 years [PPR = 0.55, 95 % CI: 0.37-0.83]. At multivariable analysis, the adjusted Prevalence Proportion Ratio of unwanted pregnancy remained high among the; sexually coerced [Adj. PPR = 2.23 95 % CI: 1.49-3.32, the unmarried [Adj. PPR = 3.38, 95 % CI: 2.31-4.93], those who had 2 pregnancies or more [Adj. PPR = 2.21, 95 % CI: 1.30-3.75], and women who experienced non consensual sexual debut [Adj. PPR = 1.72, 95 % CI: 1.16-2.54]. Similarly, the adjusted prevalence of unwanted pregnancy remained significantly lower, among women aged 20–24 years [Adj. PPR = 0.53, 95 % CI: 0.36-0.78].

**Table 3:** Prevalence of unwanted pregnancy, unadjusted and adjusted prevalence ratios among participants attending antenatal clinics in Lubaga, Uganda, 2012

Variable	Had unwanted pregnancy % (n/N)	Unadjusted PR (95 % CI)	Adjusted PR (95 % CI)
Overall^Ŧ^	18.3 (76/416)		
Sexual coercion in past twelve months			
No	17.2 (40/316)	1	1
Yes	36.0 (36/100)	2.84 (1.92-4.20)	2.23 (1.49-3.32)*
Age of participants			
15-19	27.8 (27/97)	1	1
20-24	15.4 (49/319)	0.55 (0.37-0.83)*	0.53 (0.36-0.78)*
Level of education			
Pre secondary	25.6 (20/70)	1	1
Post primary	16.6 (56/338)	0.65 (0.41-1.01)	1.06 (0.68-1.64)
Current marital status			
Married^®^	12.3 (41/334)	1	1
Unmarried^††^	42.7 (35/82)	3.48 (2.37-5.09)*	3.38 (2.31-4.93)*
Knowledge of contraceptives			
No knowledge	16.8 (19/113)	1	1
Knowledgeable	18.8 (57/303)	1.12 (0.70-1.79)	1.16 (0.77-1.76)
Contraception use at the time of conception			
No	17.6 (64/364)	1	1
Yes	23.1 (12/52)	1.31 (0.76-2.26)	1.41 (0.82-2.42)
Number of pregnancies before the current			
Prime gravid^♦^	15.1 (33/219)	1	1
1	22.2 (26/117)	1.47 (0.93-2.34)	1.44 (0.94-2.10)
2+	21.3 (17/80)	1.41 (0.83-2.39)	2.21 (1.30-3.75)*
Type of sexual debut			
Consensual	15.5 (50/323)	1	1
Non consensual	28.0(26/93)	1.81 (1.19-2.73)*	1.72 (1.16-2.54)*

In table 3 we have used row percentages that’s why the column percentages do not add up to one hundred. Unmarried ^††^ includes, single, divorced, widowed and separated. Married^®^ encompasses cohabiting and married in church. Prime gravid^♦^ means this is the first pregnancy she has never had any other before this. *Statistically significant

**Table 4:** Table of themes

Theme	Subthemes	Codes
Threatened and forced	Feared to be beaten	*He said that we had to do it [have sex] and if I shouted he would beat me.*
	Forced and used	*He told me that we had to have sex, so when I refused, he forced me and used me.*
	Feared to be abandoned	*If we don’t have sex pack your things and leave.*
	Forced to have sex by relatives	*There is one who was raped repeatedly by her uncle; he had warned her never to tell anyone.*
		*You may be staying with your sister’s husband. He will admire you and buy for you things. Then one day when your sister is not around, he will force you into sex.*
	Forced to have sex by a stranger	*I didn’t know that person though I used to see him but he raped me when I was going to bathe.*
		*A man broke into my house at night, he raped me and I became pregnant, but it took me long to discover that I was pregnant.*
Deception	Deceived and impregnated	*He deceived me that he would give me a lot of things, but when I got pregnant he left me without giving me what he promised.*
		*Ok, he first told me and I refused, then he started giving me gifts so as to seduce me and I accepted.*
	Lied with promises	*This man promised me something….I won’t tell you what it was, so we slept together, and I became pregnant. Unfortunately he never stood by his promises.*
		*Normally they deceive girls, that they will marry them, give them business or educate them; they use such things.*
		*Deceiving them that they will marry them but after using them; whether they have got HIV or pregnancy, he runs away*.
		*Gifts mostly and use of persuasive words to deceive them. A man might deceive you that I will do this for you or come at home and pick this and that.*
Poor socio-economic status	Not being able to afford	*You need something and you can’t get it from your parents, you have no money but you really want it, so you are forced to have sex with someone to get what you want.*
	Poor parents	*You find a parent leaves home in the morning without providing food for children or they will tell her that you also go and look for what you can eat.*
	Forced to have sex to get what you want	*Gifts; they tend to deceive them with gifts. Normally they give them money to buy things like clothes, powder or to plait hair. Girls normally have a list of items they want and they cannot ask everything from their parents. Therefore, gifts are so much liked by girls and they end up getting unwanted pregnancies because if you keep going to the man’s place to request for such items, he will rape you and you may become pregnant.*
		*These young girls are given money either for tuition or pocket money and they end up having sex with these men and they get unwanted pregnancy.*
Alcohol	Forced to have sex while under alcohol influence	*When a girl takes a lot of alcohol and gets drunk, men may use her and she ends up with an unwanted pregnancy and she cannot even think of contraceptives, since she will be drunk.*
		*More so when the girl takes alcohol because if a man realizes that he cannot get her when she is not drunk, he will buy a lot of alcohol for her so that she gets drunk and he will do anything he wants. That’s why they always get pregnant when they don’t know anything at all.*
	Alcohol influencing contraceptive use	*They want to go and booze but when she is drunk at night, they will be only two and the man will use her.*
		*For example If a woman is drunk, she might not really think about a condom because her reasoning has been tampered with, by the substance.*
Strategies	Youth friendly services	*If we have services specifically for young people so that there’s no fear of meeting ones mother or elder sister in the line. This will go a long way to improve the kind of service young people can access.*
	Punishing perpetrators	*What I would want the government to do is to put laws in place that punish such people who coerce girls. But because they are left unpunished they continue doing that to even more other girls. So they should really be treated by law and imprisoned so that they stop it.*
	Sex education	*By having sensitizations, teaching them how to prevent unwanted pregnancies at an early age. Sensitize them; educate them about the disadvantages of having sex at an early age. Yes, and what they watch; actually when children are there, they should not cast blue movies.*
		*Girls should be counseled and also parents themselves; they should know what a girl needs and they should always talk peacefully with their children. You have to be free with your children.*
	Youth mobile services	*May be going around distributing boxes of condoms. Yes, one will be protected with that box in the pocket. Yes, because some fear to buy them from shops.*
	Community services for the youth.	*Health workers should come to the community on specific days and just talk to us. Many of us leave with stepmothers and our fathers are too busy, so no one talks to us.*

### Types/contexts of sexual coercion and their relations with unwanted pregnancy

The most common types/contexts of sexual coercion were deception (49 %) and transactional sex (40 %), followed by physical force (34 %), verbal threats (29 %) and unwanted sexual acts (28 %). In the qualitative interviews, participants reported varied experiences of sexual coercion and circumstances under which they became pregnant. These experiences are illustrated in (Table 4) which shows the themes, sub themes and codes.

### Threats and force

Some participants reported that they became pregnant not because they were willing to do so but because; their perpetrators either verbally threatened them or forced them to have sex which resulted in their current pregnancy.

*“I was walking with my friend when a boy came and told me that so and so is calling you. The man who had sent for me was in an incomplete house, he told me that he wanted to have sex with me,…..He said that we had to do it [have sex] and if I shouted he would beat me, he forced me to have sex and some months later I realized I was pregnant” (In-Depth Interview, pregnant 17 year old).*

*“A man who had been trying to befriend me and I had refused invited me to his home to pick a gift. It rained shortly after my arrival. He told me that we had to have sex, so when I refused, he forced me and used me. After sometime, I started feeling sick and I went to hospital where they told me that I was pregnant. When I told him about it, he said that, he had not reached the time of bearing a child; and that he was not responsible for my pregnancy” (In-Depth Interview , pregnant 18 year old).*

Some KIIs also noted that some girls get raped. One of the KI’s narrated as follows:

*“Sometimes these girls are just raped and they get an unwanted pregnancy. Rape just happens; some young girls are raped by their relatives. There is one who was raped repeatedly by her uncle; he had warned her never to tell anyone. They came here with the mother and that’s when the girl told us” (KII private health facility, female nurse).*

### Deceptive sex

In another context, some women were lured to have sex through different approaches such as deceptive promises, material gifts and even drugging. Unfortunately, such women said that their partners never stood by their promises when they learnt that they had become pregnant. For example these women expressed their experiences as follows:

*“There is a man who kept on insisting that we have a relationship but I always refused, however, he kept on giving me presents. One time he invited me for a party at their home and the last thing I remember that day was that he gave me a soda; next morning when I woke up I was lying in his bed naked. Some months later I realized I was pregnant. I stopped schooling and my parents were very annoyed with me” (In-Depth Interview, pregnant 19 year old).*

*“This man promised me something….I won’t tell you what it was, so we slept together, and I became pregnant. Unfortunately he never stood by his promises” (In-Depth Interview, pregnant 20 years old).*

### Poor socio-economic status

Women also reported that they had financial challenges which partly explained their demise. Some explained that they needed finances to meet certain basics of life. However, the price to pay was an exchange for sex which resulted into their current unwanted pregnancies.

*“You need something and you can’t get it from your parents, you have no money but you really want it, so you are forced to have sex with someone to get what you want, but you may end up with an unwanted pregnancy” (In-Depth Interview, Pregnant 23 year old).*

### Similarly, some supported this finding

*“These young girls are given money either for tuition or pocket money and they end up having sex with these men and they get unwanted pregnancies” (Key Informant private health facility, female counselor).*

### Alcohol

In addition, some participants from both IDI’s and KII’s reported that some men used alcohol consumption to coerce young women into sex. They noted that alcohol interfered with one’s ability to negotiate safe sex. They also said that when one is under the influence of alcohol, it is difficult to avoid men’s coercive sexual advances. In her words, one of the girls had this to relate:

*“When a girl takes a lot of alcohol and gets drunk, men may use her and she ends up with an unwanted pregnancy and she cannot even think of contraceptives, since she will be drunk” ( In-Depth Interview, Pregnant 22 year old* ).

In all the IDI’s, young women reported to have been very angry, annoyed or to have felt so bad on realizing that they had become pregnant. One tried to abort by drinking strong tea leaves but it failed to work. Seven of the IDI participants thought about abortion but they had no money and were also concerned about the consequences of abortion.

*“When I realized I was pregnant I got very annoyed. I wanted to abort but I had no money and secondly I have seen my friends who aborted and died “ (In-Depth Interview, Pregnant 19 year old).*

### Strategies

Participants suggested strategies to counter sexual coercion and unwanted pregnancies. These included:

Health education: Most participants suggested that health education should be provided to young people less than 16 years (both boys and girls in and out of school). The teachers, health workers and parents should educate young people on how to protect themselves from sexual coercion and unwanted pregnancies.

*“Health workers should plan talks for young people. They can choose a day and come to Kawala, to give a talk after informing all young people to attend. Some of us have no one to talk to us. For example my father is a business man, he doesn’t stay at home. Others stay with stepmothers, no one gives us information. At times you may be planning to do something, but when they tell you it’s dangerous you can change your mind or start protecting yourself as you have heard by using family planning” (In-Depth Interview, pregnant 19 year old).*

Set up more youth friendly centers/corners and train providers in provision of youth friendly services: Almost all our Key Informants suggested that it was necessary to set up more youth friendly centers/corners such that young people freely access a range of reproductive health services without fear. The staff manning these centers should be properly trained to offer Youth Friendly Services (YFS).

*“You know we don’t have a system where services for young people are offered, so that young people feel comfortable to come because services are for young people only. When you send them to the family planning clinic they are going to line up with married women who will ask the young girls whether they have also come for family planning. So we need services for these young people to give them ease of access” (KII private not for profit hospital, female nurse).*

Additional strategies: Participants suggested more strategies which included imprisonment of perpetrators, using the media to air out prevention messages, adopting the unwanted children, creating jobs for young people and reducing alcohol and substance use among young people.

## Discussion

This study highlights that a quarter of our participants in our quantitative study had experienced sexual coercion in the past twelve months and nearly a third of these, had history of non consensual sexual debut. One in every six participants had an unwanted pregnancy. These findings are consistent with previous studies that have demonstrated that sexual coercion and unwanted pregnancy are common problems among young women in low income countries like Uganda [18, 19, 46].

Our results are consistent with a previous study by Koenig et al. which estimated the prevalence of sexual coercion to be 24 % in Rakai district in central Uganda. On the other hand, our findings are lower than that found by Agardh et al. 2012 [47] and Ybarra et al. 2012 [13] in western Uganda whose prevalence of sexual coercion were 31 % and 40 % respectively. The difference in prevalence estimates could be explained by the variations in the way sexual coercion was defined. For example the inclusion of being forced to expose ones sexual organ, see someone’s sexual organ, pose for a sex photo/film [47]. Our definition was narrower, and could have resulted in under estimation of sexual coercion. In spite of this, our study adds to the pool of literature that reveals that sexual coercion is a problem among young women in Uganda and provides insight into the magnitude of the problem in this urban setting in Uganda.

The commonest forms of sexual coercion were due to deception and transactional sex. Similar findings were reported by Erulkar [6] in Kenya where majority of the participants reported to have been tricked into sex. Sexual coercion has a host of negative outcomes; one of which is unwanted pregnancies [2]. Our result for the prevalence of unwanted pregnancy is within range of previous studies 10-28 % in sub Saharan Africa [48–50].

The study further shows that unwanted pregnancy was higher among the sexually coerced and those who had non consensual sexual debut. Similarly, Gessessew et al. [46] and Maharaj et al. [19] reported that young women who had been sexually coerced and those who had non consensual sexual debut respectively had higher prevalence of unwanted pregnancy. The study further revealed that sexual coercion was significantly associated with unwanted pregnancy. In Ethiopia, Garoma et al. [51] found a significant likelihood of unintended pregnancy (Unwanted and mistimed) among young women aged between 10–24 years who had been sexually coerced. Three pathways have been suggested to explain the association between sexual coercion and negative reproductive health outcomes like unwanted pregnancy [2, 18]. One pathway is that unwanted pregnancy may result directly from the act of forced sex [46]. The second pathway suggests that victims of sexual coercion are disempowered to negotiate condom /contraceptive use [18], which exposes them to unwanted pregnancy. The third pathway proposes that young women who have ever been sexually coerced, engage in risky sexual behaviors like having multiple sexual partners [6, 9] or alcohol and substance abuse [52]. These risky behaviors are interrelated with other behaviors like reduced contraceptive use and hence unwanted pregnancy [53].

We also found a significant relationship between unwanted pregnancy and non consensual sexual debut. Similar findings in Uganda [18] and South Africa [19] reported an association between non consensual sexual debut and unwanted pregnancy. Evidence suggests that, young women who have ever been sexually coerced are more likely to experience subsequent sexual coercion and are more likely to have unprotected sex [2]. A study by Koenig et al. revealed that young women who had experienced non consensual sexual debut had not used condoms on their last sexual intercourse neither had they used condoms consistently in the previous months [18]. This finding suggests the need to counsel and equip young women who have ever suffered from sexual coercion with skills to resist it, so as to protect them from subsequent sexual coercion. Perpetrators of these young women are in most cases boys or men. Therefore there is need to involve them in the fight against sexual coercion and unwanted pregnancies. Educational programs targeting young men and the old are suggested. Additionally, prevention programs on sexual coercion and unwanted pregnancy should be designed that use men to fight against perpetrators of sexual violence.

### Program implications

This study provides an insight into the magnitude of sexual coercion and its association with unwanted pregnancy among young women in urban Uganda. Strong measures to control sexual coercion and mitigate its negative outcomes among young women in this population are needed. The government should integrate comprehensive sex education into the school curriculum but also cater for the out of school youths. Sex education should be initiated at a young age before children become sexually active. Comprehensive sex education should address both the benefits of delaying sexual intercourse and also the use of contraceptives by those who are sexually active plus skills building. This will provide young people with knowledge and skills to recognize and resist sexual coercion and also to avoid its negative health outcomes like unwanted pregnancies.

It is fundamental to combine sex education with establishment of Youth Friendly Centers so that young people after receiving the health education can freely access a range of reproductive health services without fear. These services should be provided by well trained staff. There is also a need to provide free/affordable legal services for the victims, especially for those who may not be in position to afford. Mass media should be used to sensitize the community about dangers of sexual coercion and discouraging gender norms which promote sexual violence while promoting those which don’t. The legal system should ensure that perpetrators are strictly punished as stated in the Ugandan law. These programs should all be supported by policy.

### Strength and limitations

This study was well designed and used a mixed method of data collection. The qualitative method explained the quantitative findings and the in-depth interviews gave a clear picture of how these young women got sexually coerced.

However this being a cross sectional study, we couldn’t establish causality of the relationship between sexual coercion and unwanted pregnancy. Nevertheless, qualitative findings provide an understanding of the link between sexual coercion and unwanted pregnancy. Longitudinal studies are needed to establish causality between sexual coercion and unwanted pregnancy. Using young women from antenatal clinics left out young women who had been affected but were unable to attend antenatal clinics, hence limiting the generalizability of findings to the general population of young people in this community. The participants were required to recall events that happened in the past. However we think recall bias was limited because pregnancy is an unforgettable event.

## Conclusion

One in four pregnant women aged 15–24 years in this study population had been sexually coerced. Sexual coercion was significantly associated with unwanted pregnancy. Deception, transactional sex and physical force influenced unwanted pregnancies. Comprehensive sex education targeting young people (<25 years), along with availability and access to youth friendly centers may be useful in addressing sexual coercion and its negative outcomes.

## Abbreviations

IQR: Inter quartile range
SD: Standard deviation
WHO: World Health Organization
HIV: Human Immune Virus
AIDS: Acquired immunodeficiency syndrome
YFS: Youth friendly services
STI’s: Sexually transmitted infections
